# Mitochondrial transplantation attenuates hypoxic pulmonary hypertension

**DOI:** 10.18632/oncotarget.10596

**Published:** 2016-07-13

**Authors:** Liping Zhu, Jiwei Zhang, Juan Zhou, Yankai Lu, Songling Huang, Rui Xiao, Xiangyuan Yu, Xianqin Zeng, Bingxun Liu, Fangbo Liu, Mengxiang Sun, Mao Dai, Qiang Hao, Jiansha Li, Tao Wang, Tongfei Li, Qinghua Hu

**Affiliations:** ^1^ Department of Pathophysiology, School of Basic Medicine, Huazhong University of Science and Technology (HUST), Wuhan, Hubei, China; ^2^ Key Laboratory of Pulmonary Diseases of Ministry of Health, Huazhong University of Science and Technology (HUST), Wuhan, Hubei, China; ^3^ Department of Pathology, Union Hospital, Huazhong University of Science and Technology (HUST), Wuhan, Hubei, China; ^4^ Department of Pathology, Tongji Hospital, Huazhong University of Science and Technology (HUST), Wuhan, Hubei, China; ^5^ Department of Respiratory and Critical Care Medicine, Tongji Hospital, Tongji Medical College, Huazhong University of Science and Technology (HUST), Wuhan, Hubei, China; ^6^ Department of Pathology, School of Basic Medical Sciences, Hubei University of Medicine, Shiyan, China; ^7^ Department of Clinical Laboratory of Xuzhou Central Hospital, Xuzhou, China

**Keywords:** hypoxia, mitochondria, transplantation, pulmonary hypertension, vasoconstriction, Pathology Section

## Abstract

Mitochondria are essential for the onset of hypoxia-induced pulmonary vasoconstriction and pulmonary vascular-remodeling, two major aspects underlying the development of pulmonary hypertension, an incurable disease. However, hypoxia induces relaxation of systemic arteries such as femoral arteries and mitochondrial heterogeneity controls the distinct responses of pulmonary versus femoral artery smooth muscle cells to hypoxia *in vitro*. The aim of this study was to determine whether mitochondrial heterogeneity can be experimentally exploited in vivo for a potential treatment against pulmonary hypertension. The intact mitochondria were transplanted into Sprague-Dawley rat pulmonary artery smooth muscle cells *in vivo* via intravenous administration. The immune-florescent staining and ultrastructural examinations on pulmonary arteries confirmed the intracellular distribution of exogenous mitochondria and revealed the possible mitochondrial transfer from pulmonary artery endothelial cells into smooth muscle cells in part through their intercellular space and intercellular junctions. The transplantation of mitochondria derived from femoral artery smooth muscle cells inhibited acute hypoxia-triggered pulmonary vasoconstriction, attenuated chronic hypoxia-induced pulmonary vascular remodeling, and thus prevented the development of pulmonary hypertension or cured the established pulmonary hypertension in rats exposed to chronic hypoxia. Our findings suggest that mitochondrial transplantation possesses potential implications for exploring a novel therapeutic and preventive strategy against pulmonary hypertension.

## INTRODUCTION

Pulmonary hypertension is a lethal complication of patients with chronic lung diseases who usually experience hypoxemia. It is generally accepted that hypoxia-induced pulmonary hypertension (HPH) is attributed to pulmonary vasoconstriction and pulmonary vascular remodeling [1, 2, 3].

Mitochondria are critical in the initiation of both hypoxia–induced pulmonary vasoconstriction (HPV) and pulmonary vascular remodeling (HPR). By contrast, hypoxia triggers relaxation in systemic vessels [4, 5]. Mitochondria in pulmonary artery smooth muscle cells (PASMCs) appear structurally and functionally distinct from those in systemic arteries [6, 7]. Our recent study employing cross transplantation of mitochondria between PASMCs and femoral artery smooth muscle cells (FASMCs) revealed that mitochondrial heterogeneity critically controls the distinct responses of pulmonary versus femoral artery under hypoxia [8]. We found that the transplantation of mitochondria derived from FASMCs inhibited hypoxia-induced cell membrane potential depolarization, [Ca2^+^]_i_ elevations in PASMCs and attenuated hypoxia-triggered constriction of pulmonary arteries *in vitro* [8]. We therefore performed the current study to explore whether mitochondria can be transplanted into pulmonary arteries *in vivo*, and if so whether the transplantation of mitochondria prepared from FASMCs can be experimentally exploited to treat HPH and whether the effectiveness of mitochondrial transplantation is mechanistically associated with their modulation of HPV and HPR.

Here we showed that isolated mitochondria can be delivered or transplanted into pulmonary arteries *in vivo*. We found that the transplantation of mitochondria derived from FASMCs inhibited acute hypoxia-induced pulmonary vasoconstriction, restrained pulmonary vascular remodeling and pulmonary hypertension in rats exposed to chronic hypoxia. Our *in vivo* study provides additional and indispensable evidence for the determinant role of mitochondria in pulmonary vascular responses to hypoxia and the potential significance of the enforced relocation of mitochondria in exploring a novel therapy and prevention against pulmonary hypertension.

## RESULTS

### Transplantation of exogenous mitochondria into pulmonary arteries by intravenous administration

**Figure 1:**
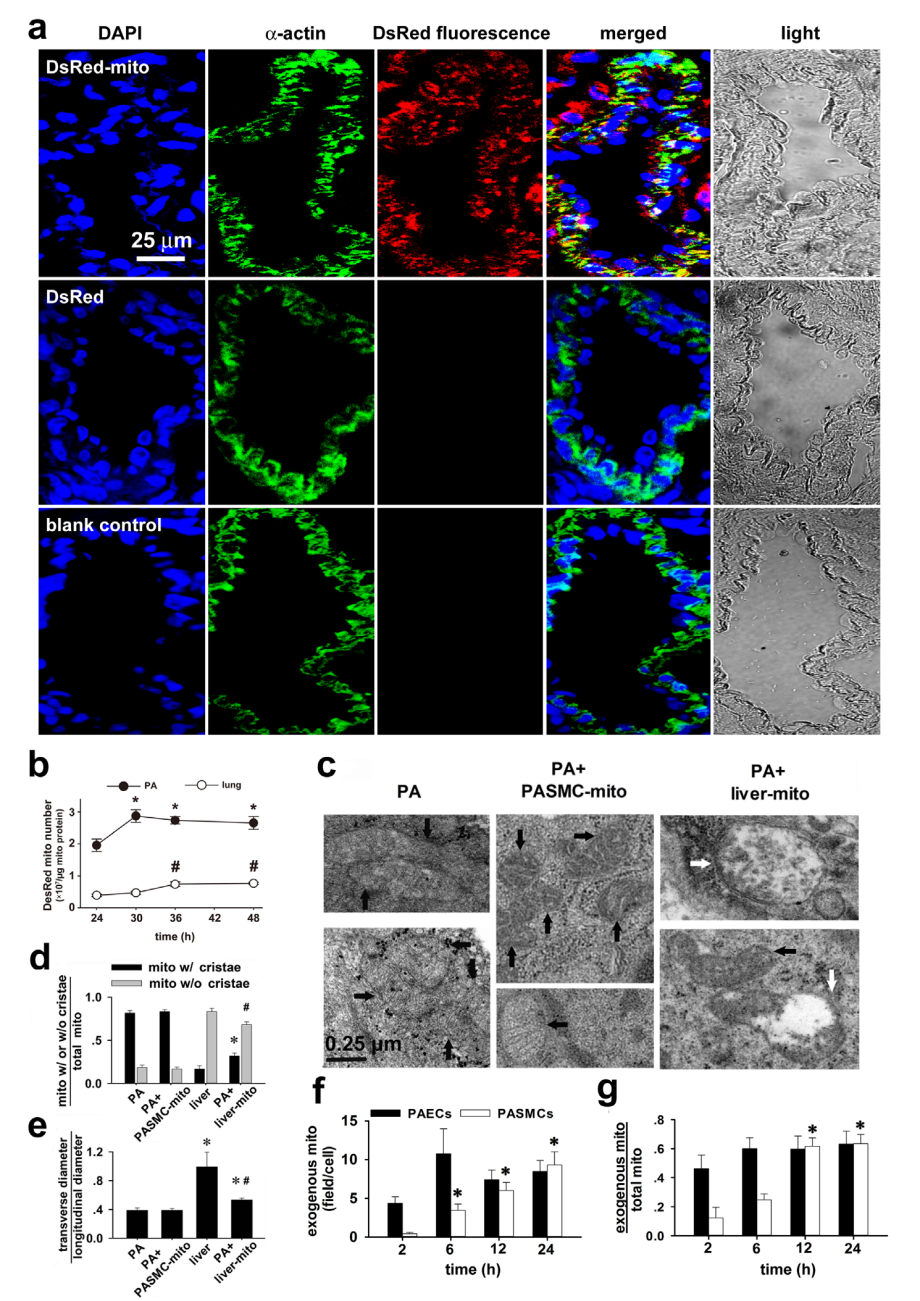
Transplantation of mitochondria into pulmonary arteries by intravenous administration **a** Immunohistochemical stainings of lung tissue in rats after intravenous injection of DsRed-labeled mitochondria (*upper*), DsRed- (*middle*) and in control (*lower*) showing DAPI (blue), smooth muscle cell marker α-actin (green), DsRed (red), overlap and light field (n=3 for each). **b**. Flow cytometry analysis of the change of mitochondrial number over time in pulmonary arteries (PA) and lung tissues in rats 24-48 hours after intravenous administration of DsRed-labeled mitochondria (* # *p* < 0.05 *vs* 24 hour, n=3 for each). **c**-**e** Electron micrographs showing mitochondria (mito) in PASMCs *in vivo* in control rats (black arrows, *left*, c) and rats after intravenous injection of PASMC-derived mitochondria (PASMC-mito) (black arrows, *middle*, c), mixture of mitochondria with distinct morphology in PASMCs *in vivo* after intravenous introduction of the liver mitochondria (liver-mito) (black and white arrows, *right*, c) and quantitation of two shapes of mitochondria (* # *p* < 0.05 *vs.* mito with/without cristae in PASMCs, respectively, d) as well as their ratio of width to length (* *p* < 0.05 *vs.* PASMCs, # *p* < 0.05 *vs.* liver, e). Quantitation obtained from 115, 158, 232 and 166 mitochondria of 24, 17, 20 and 31 cells from 4 separate rats for PASMCs, PASMCs injected with PASMC-mito, Wilson's liver cells and PASMCs injected with liver-mito, respectively. **f**-**g** PAs were isolated at 2, 6, 12 and 24 hours after intravenous administration of Wilson's liver mitochondria, respectively and subject to ultrastructure examination. Averaged mitochondria quantity per view (f, ~ 3×3μM^2^) and the ratio of liver mitochondria of total mitochondria (g) were obtained from 9 to 12 individual pulmonary artery endothelial cells (PAECs) and 14 to 21 individual pulmonary artery smooth muscle cells (PASMCs) from 3 separate animals for each time point, respectively (* *p* < 0.05 *vs*. 2 hours).

To determine whether mitochondrial transplantation is applicable *in vivo*, DsRed-labeled mitochondria of FASMCs were intravenously injected into rats, the subsequent immunohistochemical stainings in lung tissues using antibody against DsRed or SMC specific α-actin showed the overlapping of the majority of DsRed with α-actin, indicating delivery of exogenous mitochondria into PASMCs (Figure 1a). DsRed was also noted in a few cells surrounding PASMCs. Additionally, DsRed was found in some area directly connected with, however not overlapping with α-actin, indicating the possible adhesion or attachment of exogenous mitochondria at the surface of PASMCs (Figure 1a). Flow cytometry analysis of mitochondria isolated from pulmonary arteries and lung tissues in the rats showed that DsRed-labeled, exogenous mitochondria number in pulmonary arteries increased from ~ 1.96×10^7^/μg mitochondrial protein at 24 hours after intravenous administration of mitochondria to ~ 2.87×10^7^/μg mitochondrial protein at 30 hours after administration and then remained relatively constant for up to an additional 18 hours (Figure 1b). The exogenous mitochondria number in the lung tissue were ~ 0.39×10^7^/μg mitochondrial protein at 24 hours after intravenous administration of mitochondria and increased to ~ 0.74×10^7^/μg mitochondrial protein at 36 hours after administration and then remained relatively constant for up to an additional 12 hours (Figure 1b). The intravenous administration of DsRed protein at the concentration of 0.76 μg/ml, yielding fluorescent intensity equal to 2.25×10^8^/ml DsRed-labeled mitochondria used, did not result in any internalization of DsRed within PASMCs (Figure 1a, middle), indicating that DsRed-labeled mitochondria transplanted into PASMCs were intact organelles rather than merely endocytosed DsRed protein aggregates.

To confirm the above finding, mitochondria from Wilson's disease rat liver were introduced intravenously and then pulmonary arteries were fixed for ultrastructure examination. In smooth muscle cells of pulmonary arteries isolated from control rats and rats with intravenous injection of PASMCs-derived mitochondria, mitochondria were long spindle and their cristae were clear (Figure 1c, left and middle). In smooth muscle cells of pulmonary arteries isolated from rats with intravenous injection of mitochondria from Wilson's disease rat liver, mitochondria were mixture of oval, round and spindle ones with clear, unclear, swelling or disappeared cristae (Figure 1c, right and 1d-1e).

To find out more about the mechanism of the transfer of mitochondria from the blood stream, the Wilson's liver mitochondria were quantified in pulmonary arteries isolated at a series of time points after mitochondria administration by vein. The dynamic of transplantation of exogenous mitochondria into pulmonary artery endothelial cells (PAECs) was different from PASMCs. The transplantation of exogenous mitochondria into PAECs occurred as early as 2 hours after administration and appeared to reach a steady state level at 6 hours (Figure 1f-1g). For PASMCs, however, the transplantation of exogenous mitochondria occurred from 2-6 hours after administration and saturated at 12-24 hours (also Figure 1f-1g). The time lapse between exogenous mitochondria transplantation into PAECs and PASMCs may indicate that mitochondria can transfer into smooth muscle cells at least in part through the endothelium.

**Figure 2:**
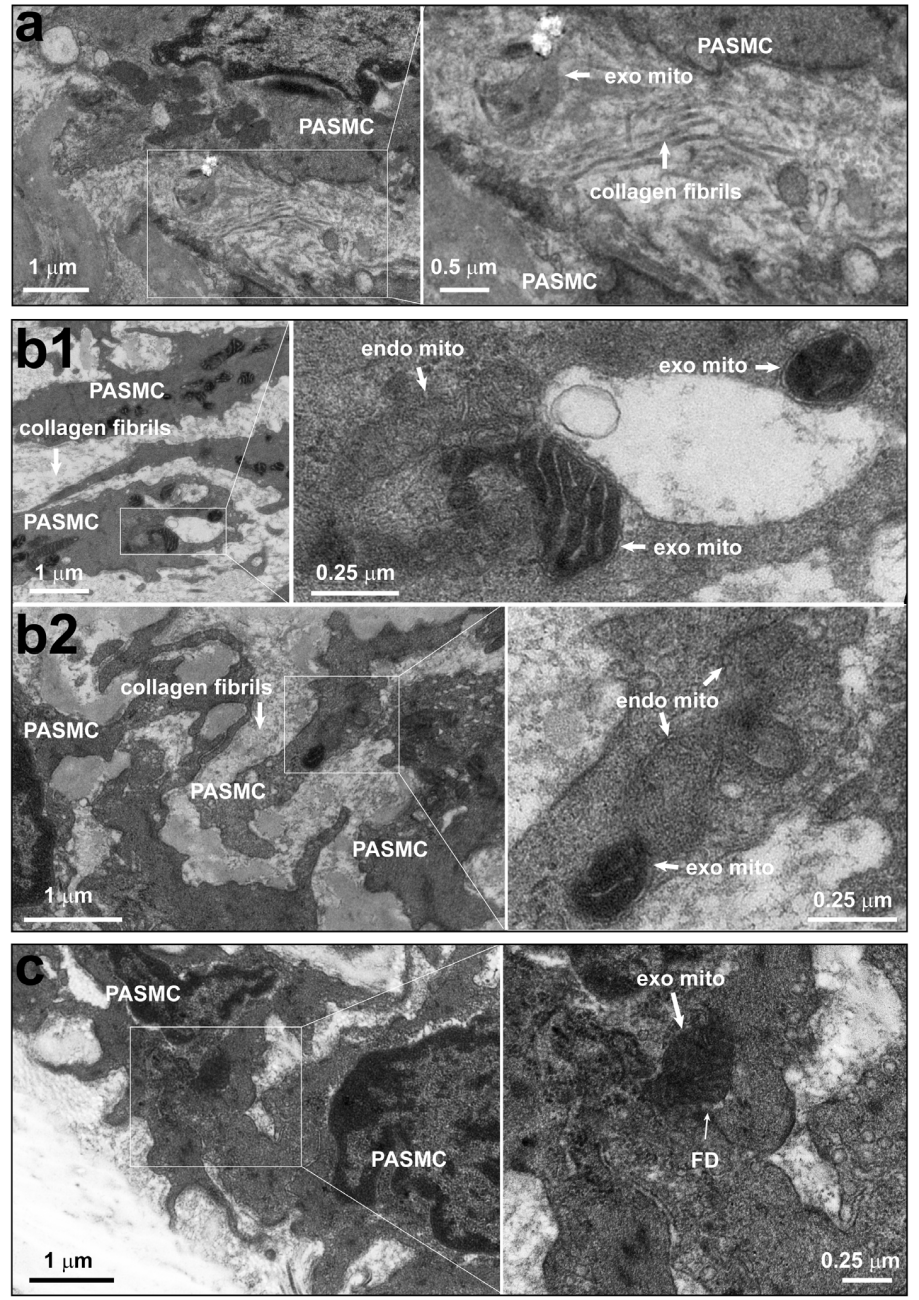
The existence of individual mitochondria within the intercellular space and junction between pulmonary artery endothelium and smooth muscle cells **a**. Rats were intravenously administrated with mitochondria prepared from Wilson's disease rat liver, which were round with swelling, unclear or disappeared cristae, and then pulmonary arteries were isolated for ultrastructural examination. Representative EM imagings showed one mitochondrion within the intercellular space (a). Out of a total 37 fields with simultaneous existence of endothelial cells and smooth muscle cells from 8 separate pulmonary artery preparations, 9 mitochondria which were round without clear cristae in 7 fields, no mitochondria in the remaining fields were identified within the intercellular space. **b**.-**c**. Rats were intravenously administrated with ascorbate peroxidase, APEX-labeled mitochondria prepared from femoral artery smooth muscle cells, then pulmonary arteries were isolated for ultrastructural examination. Representative EM imagings showed the intracellular localization of APEX-labeled, exogenous mitochondria with apparent contrast only in the mitochondrial matrix, not the intermembrane space (b1 and b2); the “on-going” navigation (*top right area* in the enlarged frame of b1, and b2) and “accomplished” entry (*middle area* in the enlarged frame of b1) of APEX-labeled, exogenous mitochondria from intercellular space into a smooth muscle cell; and one APEX-labeled, exogenous mitochondrion crossing through a focal discontinuity between endothelial cell and smooth muscle cell (c), the featured myoendothelial junctions allowing bi-directional signaling between endothelial cells and smooth muscle cells in pulmonary arteries. Out of a total of 78 cells examined, 326 and 26 APEX-labeled mitochondria were identified within and crossing into the cytosol, respectively (PAEC, pulmonary artery endothelial cell; PASMC, pulmonary artery smooth muscle cell; FD, focal discontinuity; exo mito, exogenous mitochondria; endo mito, endogenous mitochondria).

In experiments using Wilson's liver mitochondria or engineered ascorbate peroxidase (APEX)-labeling as mitochondrial tracer [8, 9, 10] we determined the borderlines of adjacent cells under lower amplification and then examined the area(s) under high amplification including the collagen fibrils in the intercellular space. Our ultrastructural examination unexpectedly identified the localization of individual mitochondria within the intercellular space between pulmonary artery endothelial cells and smooth muscle cells (Figure 2a), the “ongoing”-like and “accomplished”-like uptake of exogenous mitochondria into smooth muscle cells (Figure 2b). Also occasionally identified were individual mitochondria “traveling” through the “focal discontinuities” (Figure 2c), a featured intercellular junction between pulmonary artery endothelial cells and smooth muscle cells as previously reported [11, 12, 13, 14]. Our ultrastructural examination also identified more exogenous mitochondria within superficial layers of smooth muscle cells than deep layers of smooth muscle cells in pulmonary arteries (~ 0.7 μm underneath endothelium in Figure 2b1 vs. ~ 11.5 μm in Figure b2). These results provide a clue for the mitochondrial transfer from pulmonary artery endothelial cells into smooth muscle cells at least in part through their intercellular space and featured junctions. Of note, we observed the distribution of exogenous mitochondria within intercellular space at 0.5-12 hours, not 24 hours after intravenous injection.

**Figure 3:**
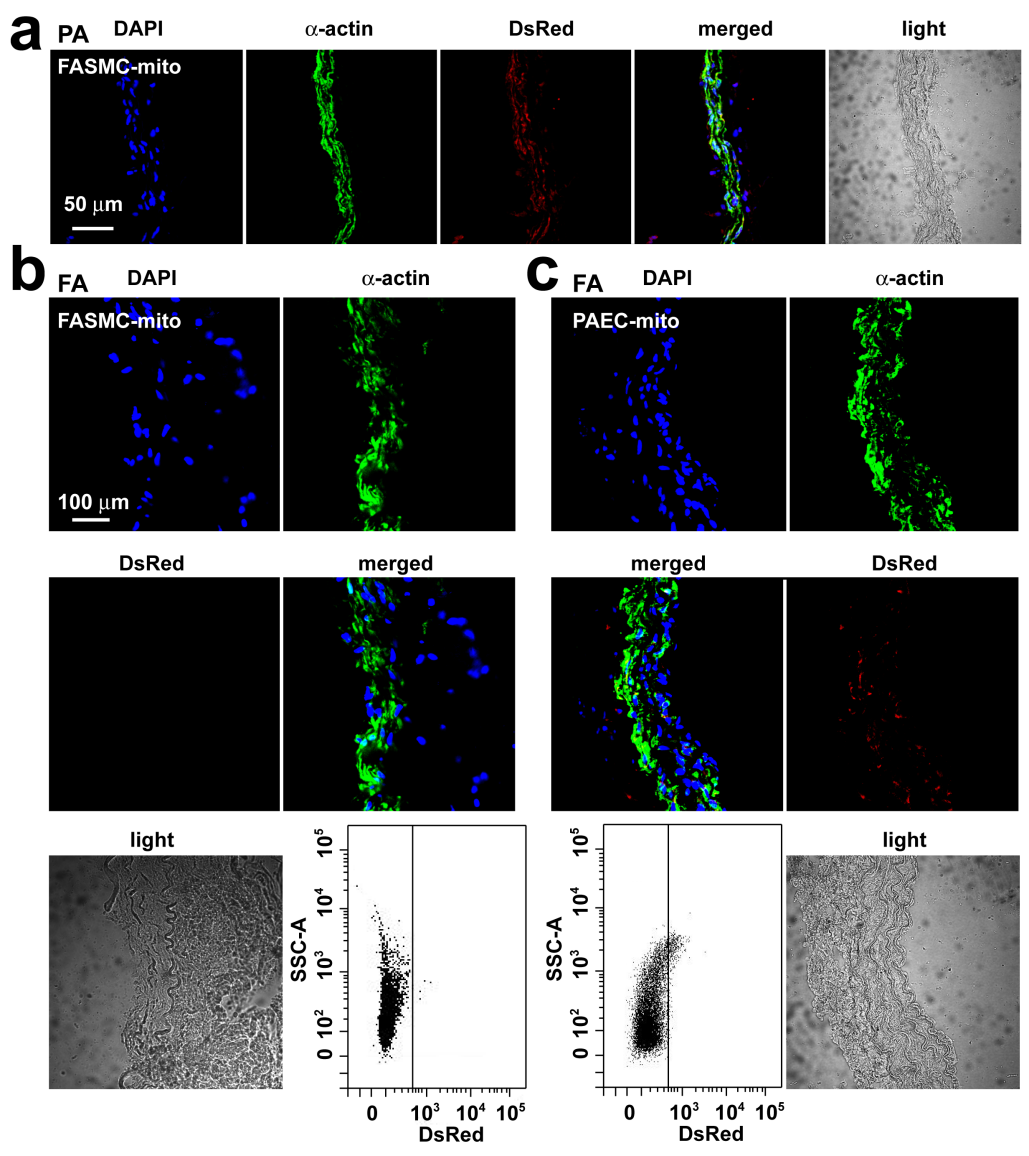
Transplantation of small mitochondria into femoral arteries by intravenous administration **a.** Immunohistochemical stainings of pulmonary artery (PA) in rats after an intravenous injection of DsRed-labeled mitochondria prepared from femoral artery smooth muscle cells (FASMC-mito, width ~296 nm) showing DAPI (blue), SMC marker α-actin (green), DsRed (red), overlap of the above three (merged) and light field. **b**.-**c**. Immunohistochemical stainings of femoral artery (FA) in rats after an intravenous injection of DsRed-labeled FASMC-mito (width ~296 nm, b) or DsRed-labeled mitochondria prepared from pulmonary artery endothelial cells (PAEC-mito, width ≤ 150 nm, c) showing DAPI (blue), SMC marker α-actin (green), DsRed (red), overlap of the above three (merged) and light field, and the representative flow cytometry for the separation of DsRed-labeled exogenous mitochondria from PA. The mitochondria isolated from FA of rats without mitochondrial injection were used as a negative control to set up voltages of side scatter (SSC) for flow cytometry analysis. Each represents stainings of 9 artery segments from 3 animals.

**Figure 4:**
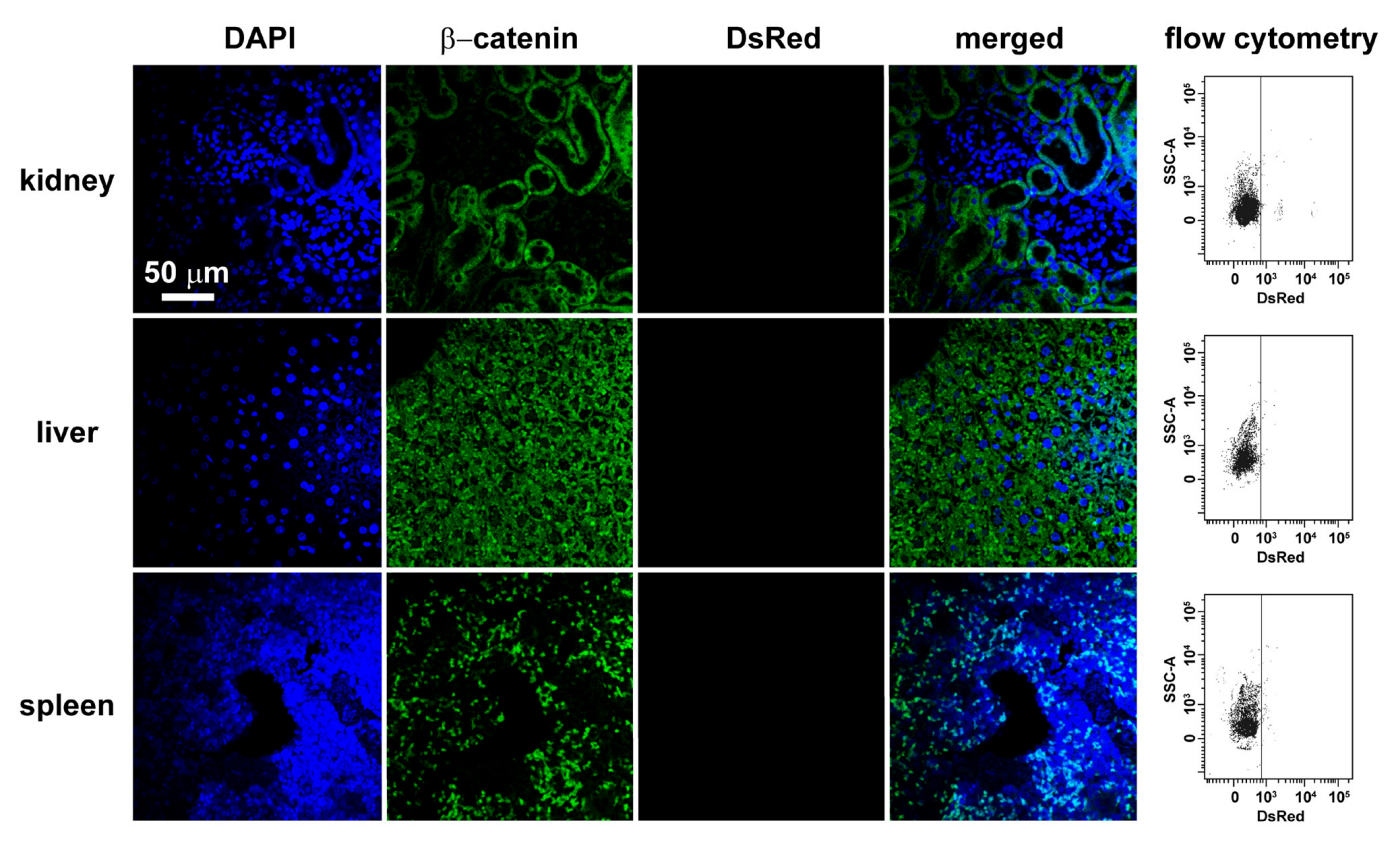
The localization of small amount of mitochondria in kidney, liver and spleen after intravenous injection Immunohistochemical stainings of kidney, liver and spleen in rats after intravenous injection of DsRed-labeled mitochondria showing DAPI (blue), β-catenin as a cell membrane marker (green), DsRed (red), or overlap of the above three and light field. Each organ was cut through an incision along the midline of sagittal plane and two separate sections were sampled at the hilus and periphery region adjacent to capsule for each organ, respectively. All the fields of each section were examined under fluorescent microscope. Shown are representative stainings of 4-5 separate fields photographed randomly from each sliced sections of two individual rats for each organ. Also shown for each organ are the representative flow cytometry for the separation of DsRed-labeled exogenous mitochondria from kidney, liver and spleen, respectively. The mitochondria isolated from rats without mitochondrial injection were used as a negative control to set up voltages of side scatter (SSC) for flow cytometry analysis.

Only very small amount of DsRed-labeled mitochondria of FASMCs (FASMC-mito, averaged width ~ 296 nm) identified by flow cytometry to be localized in femoral arteries after intravenous administration (*lower*, Figure 3b), the amount of them appeared too few to be detectable by histoimmunostaining (*upper*, Figure 3b). However, exogenous mitochondria were identified by both flow cytometry and histoimmunostaining to be localized within smooth muscle cells in femoral arteries of rats injected with DsRed-labeled small mitochondria prepared from PAEC (PAEC-mito at 2.25×10^8^/ml, width ≤ 150 nm selected and enriched with a Millipore Durapore membrane filter, 150 nm) (Figure 3c). The small amount of DsRed-labeled FASMC-mito was also identified by flow cytometry to be also localized in other end organs of kidney, liver and spleen (Figure 4), the quantity of them however was apparently too few to be detected by histoimmunostaining (Figure 4). These results support the speculation that the majority of FASMC-mito transduced the pulmonary artery wall not the systemic wall through a mechanism associated with mitochondrial size.

### Attenuation of HPV, HPR and HPH by intravenously injected mitochondria

**Figure 5:**
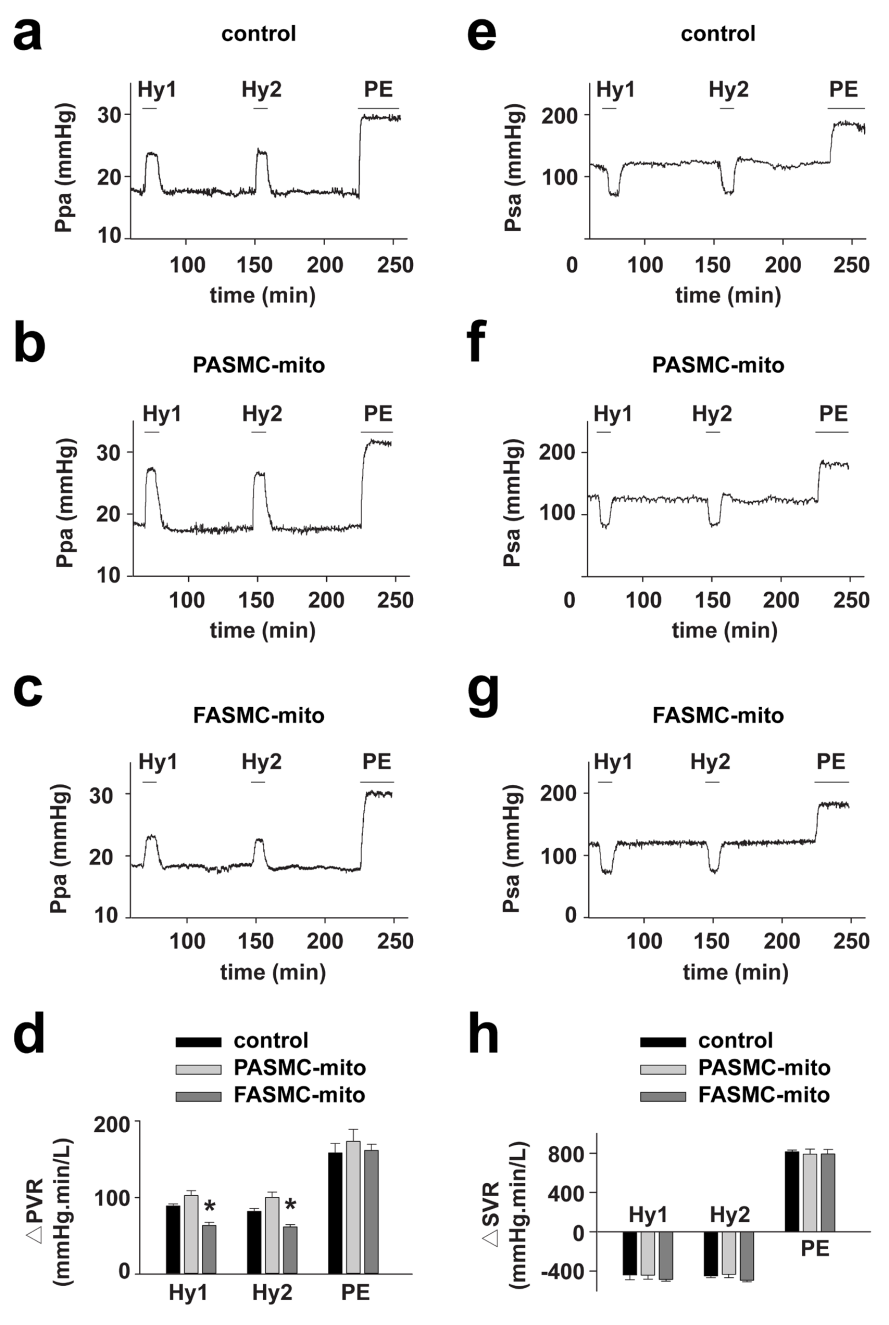
Attenuation of acute hypoxia-induced pulmonary vasoconstriction in rats by transplanted mitochondria via intravenous injection Hemodynamic monitoring the pressure of pulmonary artery (Ppa) and the pressure of systemic artery (Psa) showing pulmonary (**a**-**d**) and systemic vascular responses (**e**-**h**) to hypoxia and PE in control rats (a and e), rats intravenously injected with mitochondria from PASMCs (PASMC-mito) (b and f) or FASMCs (FASMC-mito) (c and g) and summary (n=3-5, * *p* < 0.05 *vs*. control or PASMC-mito, respectively, d and h).

Hypoxia successively triggered typical HPV (Figure 5a) and relaxation of systemic artery in rats (Figure 5e). HPV was found to be slightly augmented in rats injected with mitochondria derived from PASMCs (Figure 5b and 5d), and significantly attenuated in rats injected with mitochondria from FASMCs (Figure 5c-d). The hypoxia-induced relaxation of systemic artery was not affected by intravenous injection of mitochondria derived from either PASMCs or FASMCs (Figure 5e-5h).

**Figure 6:**
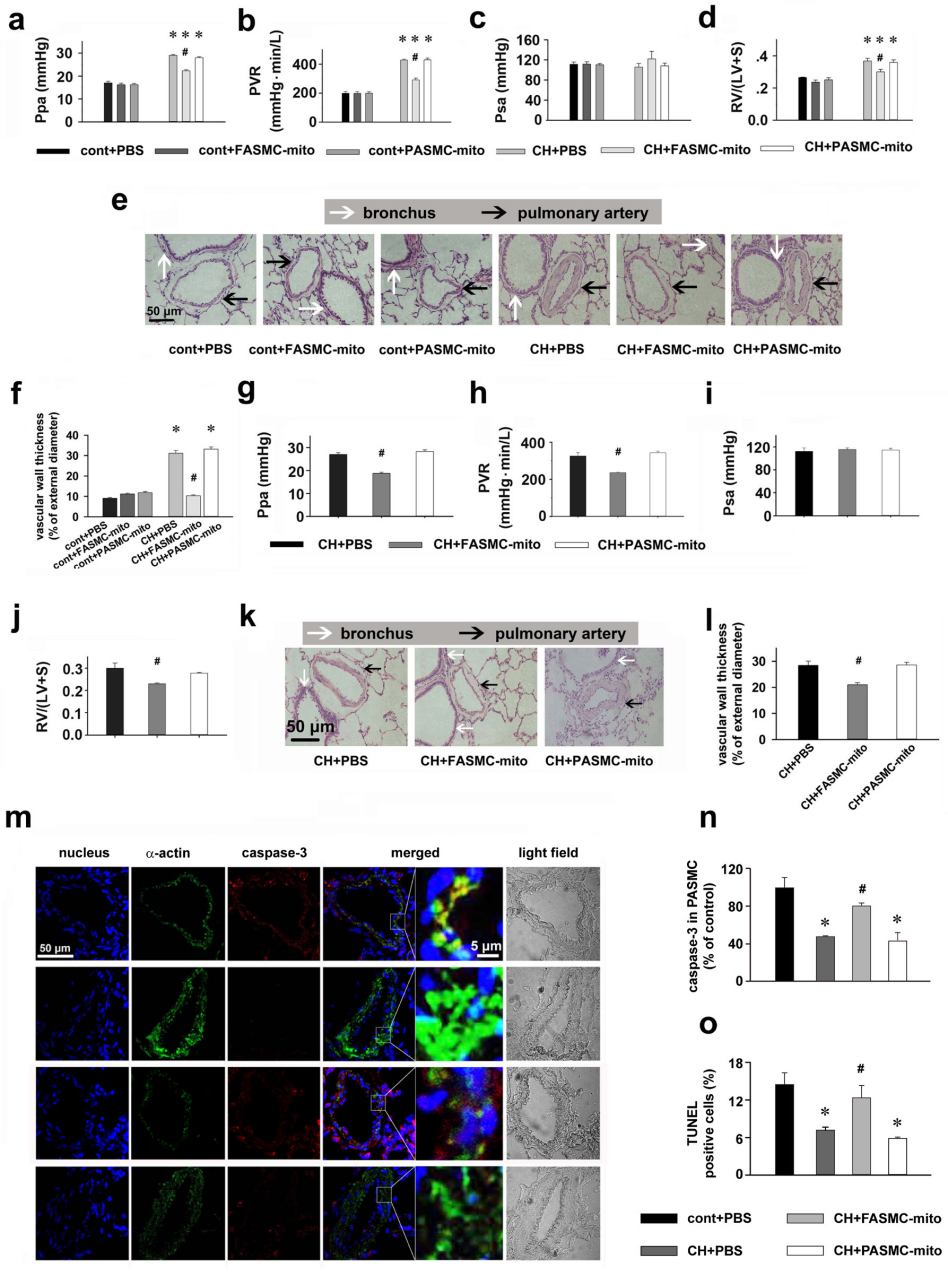
Therapeutic and preventive effects of transplanted mitochondria via intravenous injection on chronic hypoxia-induced pulmonary hypertension in rats **a-l.** Alterations of Ppa (a, g), pulmonary vascular resistance (PVR, b, h), Psa (c, i), index of right ventricular hypertrophy (d, j), representative HE stainings (e, k) and summary of pulmonary artery muscularization (f, l) in control (cont) rats or rats exposed to chronic hypoxia (CH), with intravenous injections of PBS, FASMC-mito or PASMC-mito after hypoxia (n=4, a-f), or during the last 2 weeks of hypoxic exposure (n=4, g-l). **m-n.** Representative immuohistochemical stainings of nucleus, smooth muscle α-actin and active caspase3 (m) and summary of apoptosis in pulmonary arteries in control rats, rats chronically exposed to hypoxia and injected with PBS, FASMC-mito or PASMC-mito (n=6, n; n=5, o). * *p* < 0.05 *vs*. control, # *p* < 0.05 *vs*. hypoxia-exposed rats injected with PBS or PASMC-mito, respectively.

Chronic hypoxia increased pulmonary artery pressure, pulmonary artery resistance, Fulton's index and pulmonary artery muscularization (Figure 6a-6f), which were significantly attenuated in rats injected with mitochondria derived from FASMCs either after (Figure 6a-6f) or during hypoxic exposure(Figure 6g-6l), but not in rats injected with mitochondria from PASMCs (Figure 6a-6l). Intravenous introduction of mitochondria did not affect systemic pressure (*p* = NS *vs*. control, Figure 6c and 6i). As shown in Figure 6m-6o, chronic hypoxia inhibited the apoptosis of PASMCs *in vivo*, the key step for HPR and HPH [2, 15]. The process, however, was significantly attenuated by intravenous injection of exogenous mitochondria from FASMCs, not PASMCs (Figure 6m-6o). Thus, therapeutic and preventive potential of exogenous mitochondria from FASMCs against HPH seems associated with attenuating acute hypoxia-induced pulmonary vasoconstriction and chronic hypoxia-induced inhibition of apoptosis of PASMCs and the subsequent vascular remodeling.

### Functions of transplanted mitochondria in pulmonary arteries *in vivo*

**Figure 7:**
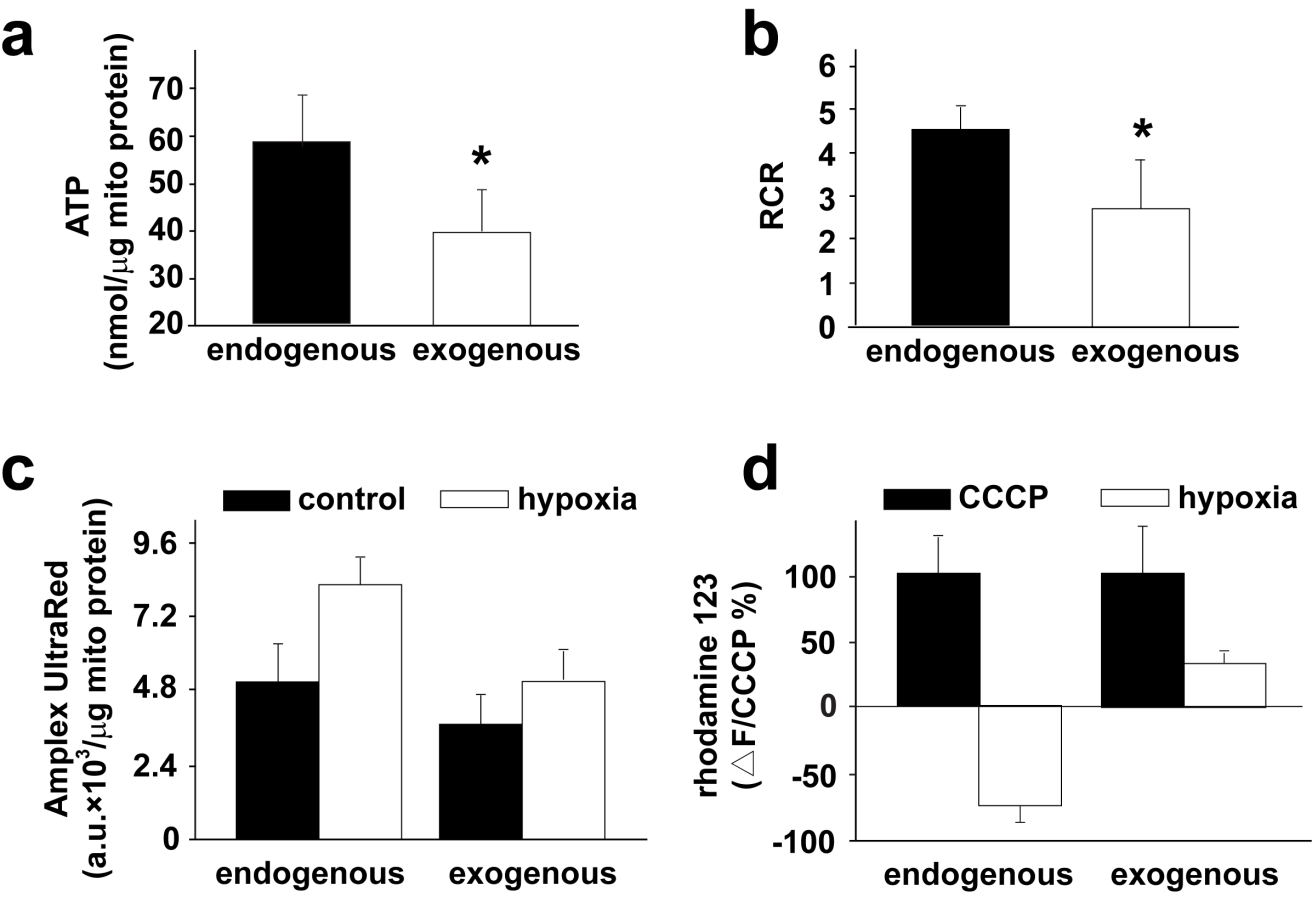
Functions of transplanted mitochondria in pulmonary arteries *in vivo* **a.-d.** Rats were intravenously injected with 2.25×10^8^/ml GFP-labeled mitochondria were isolated from 6 mg, wet weight)subjected to flow cytometry for the subsequent sorting and recovery of the endogenous, unlabeled mitochondria and the exogenous, GFP-labeled mitochondria. The recovered mitochondrial were evaluated for their ability to produce ATP (a), respiratory control ratio (RCR, b), their capability to generate ROS under hypoxia, respectively (c) and their changes of MMP in response to hypoxia (d). * *p* < 0.05, 3 PAs from 3 separate rats for each.

To determine if exogenous mitochondria are functional *in vivo* as they were after transplantation into PASMCs in culture and into pulmonary arteries *in vitro* [8], we recovered mitochondria after their transplantation into pulmonary arteries in rats. Mitochondria were isolated from pulmonary arteries in rats after intravenous injection of the GFP-labeled then subjected to flow cytometry for sorting and recovery of the endogenous as well as GFP-labeled, exogenous mitochondria. Functional evaluations showed that the exogenous mitochondria retained their ability to generate ATP and their ability to produce ATP was lower than endogenous mitochondria; the exogenous mitochondria held lower respiratory control ratio (RCR) and produced less amount of H_2_O_2_ under hypoxic condition as compared to endogenous mitochondria; the basal level of MMP were similar in exogenous and endogenous mitochondria, hypoxia induced depolarization of MMP in exogenous mitochondria, however hyperpolarization in endogenous mitochondria (Figure 7a-7d).

### Specific effect of transplanted mitochondria on pulmonary hypertension (PH)

**Figure 8:**
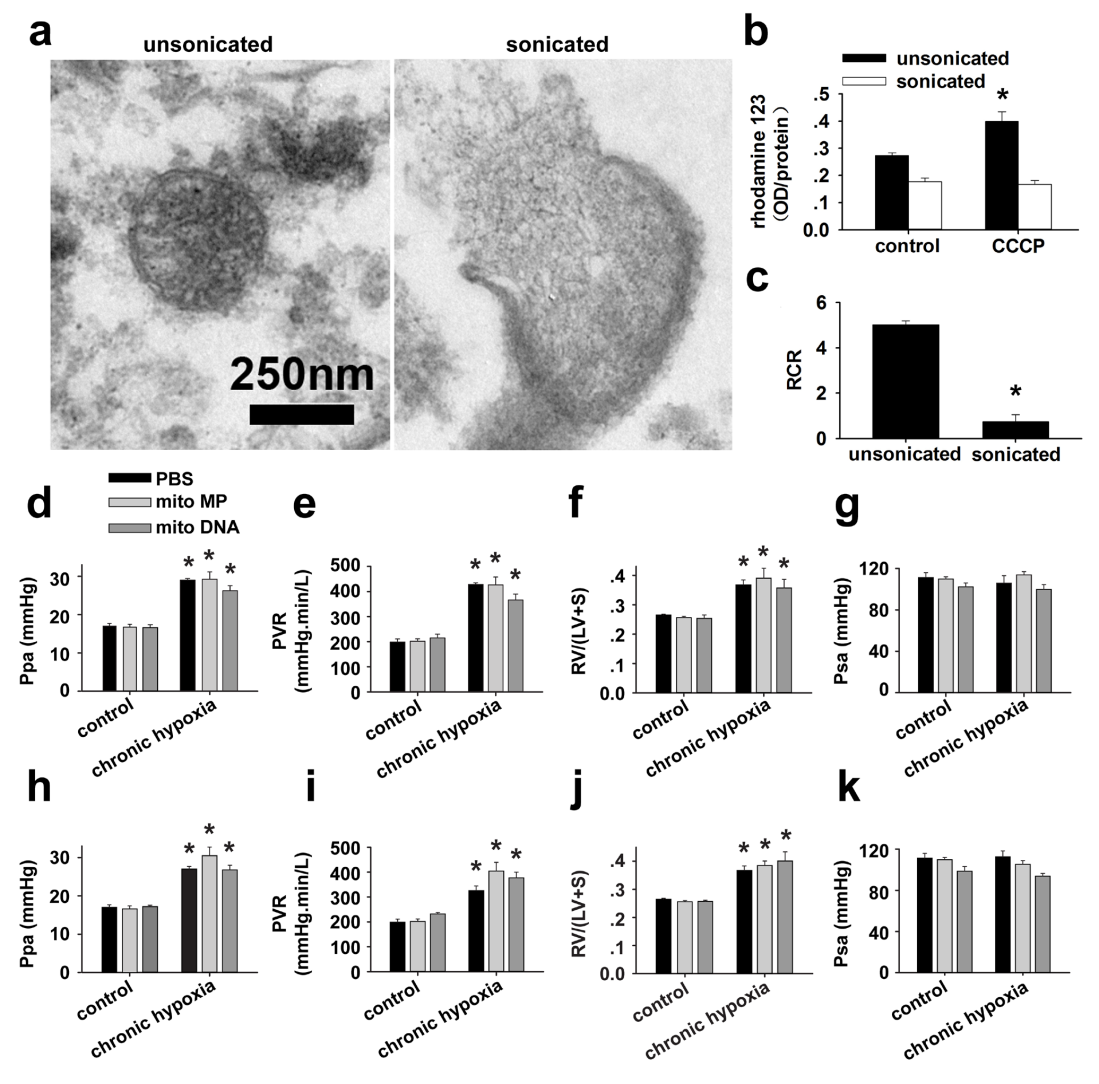
Specific effects of transplanted mitochondria **a-c.** Isolated mitochondria were either unsonicated or sonicated with an ultrasonic processor (130 watt, 20 kHZ, Amp1 30%) for three times, each for 15 sec, then subjected to ultrastructural examination and functional test of mitochondrial membrane potential (MMP) and respiratory control ratio (RCR). a. Representative images showing an intact mitochondrion with double-membrane and clear cristae from unsonicated preparation (*left*), a broken and swollen mitochondrion with discontinuous membrane, efflux of mitoplasts and disappeared cristae from sonicated preparation (*right*). b. 10 μM CCCP induced an increase in rhodamine123, indicating depolarization in MMP in unsonicated mitochondria preparation (* *p* < 0.05, n=3), however failed to induce any response in sonicated mitochondria preparation (*p*=NS, n=3). c. Sonication almost completely damped the respiratory function of mitochondria (* *p* < 0.05, n=3 for each). **d.-k.** Hemodynamic monitoring the pressure of pulmonary artery (Ppa, d and h), pulmonary vascular resistance (PVR, e and i), index of right ventricular hypertrophy (RV/(LV+S), f and j) and the pressure of systemic artery (Psa, g and k) in control rats and rats after exposure to 4 weeks of chronic hypoxia. Rats were intravenously injected with either PBS, mitochondrial particles (MPs) prepared by sonication of isolated mitochondria from FASMCs (FASMC-MP) or mitoDNA every other day for two weeks either after the 4 weeks of hypoxia (d-g) or from the third week of hypoxia exposure (h-k) (n=3-5 for each, * *p* < 0.05 *vs*. control).

To further verify if the attenuation of PH by FASMC-mito is dependent on mitochondrial transfer into PASMCs and not on an unspecific side effect of mitochondrial particles (MPs) or mitochondrial DNA (mitoDNA) in the blood stream, MPs prepared by sonication and isolated mitoDNA were administrated into rats. Hemodynamic monitoring revealed that the administration of mitoDNA or MPs without intact ultrastructure and respiratory function (Figure 8a-8c), either after (Figure 8d-8g) or during (Figure 8h-8k) the four weeks of exposure to hypoxia did not affect the development of chronic hypoxia-induced PH.

## DISCUSSION

The mechanism(s) involved in transplantation of exogenous mitochondria into smooth muscle cells of pulmonary arteries may be more complicated *in vivo* than *in vitro* as reported recently from other lab [16] and ours [8]. In addition to the direct interaction between mitochondrial membrane and cytoplasma membrane and the subsequent processes like fusion and or endocytosis as suggested from recent works of another lab [16] and ours [8], the other possible pathways may be related with the interspace and gap junctional channels between endothelial and smooth muscle cells as well as those between smooth muscle cells, since mitochondrial transfer between cells was reported in very recent studies [17, 18]. Our ultrastructural examinations on pulmonary vasculature identified the featured myoendothelial junctions, “focal discontinuities” (Figure 2). Previous studies on pulmonary vasculature in different species including rat and mouse have well established that the cell membrane is focally discontinuous in the internal elastic lamina at the intercellular junctions achieved at projections from either the endothelial cell or the smooth muscle cell or both [11, 12, 13, 14]. These featured intercellular junctions, named as “focal discontinuities” hold a width of 0.5-1 μm, a size much larger than the averaged width of the mitochondria < 0.3 μm employed in the current study, allowing direct cell-cell contact and facilitating intercellular exchange [11, 12, 13, 14]. Furthermore, we occasionally found the existence of individual mitochondria around or “traveling” through the focal discontinuities and the intercellular space (Figure 2). These results might provide a clue for the mitochondrial transfer from pulmonary artery endothelial cells into adjacent smooth muscle cells and deep layers of smooth muscle cells at least in part through their intercellular space and or junctions. This might also be the reason for the transplantation of exogenous mitochondria into a few cells surrounding pulmonary artery SMC (Figure 1). Of note, the intravenous injection resulted in transplantation of exogenous mitochondria into pulmonary, not systemic arteries (Figure 3). This is possibly because of the molecular sieve of lung, the pharmacological concept. The size of the mitochondria prepared from SMCs in the present study was ~ 296 nm in width [8]. The nano lipid particles with diameter size of this range intravenously administrated have been well determined to distribute or retain mainly in the organs of the reticulo-endothelial system including lung [19, 20, 21]. Similarly, a liposome-based delivery system has been successfully employed to introduce siRNA into pulmonary vasculature *in vivo* by intravenous injection [22, 23]. The intracellular delivery of DsRed-labeled small mitochondria prepared from pulmonary artery endothelial cells (≤150 nm) into smooth muscle cells in femoral arteries of rats after an intravenous injection seems to support this speculation (Figure 3).

The intracellular delivery of mitochondria was successfully achieved in previous studies by direct intracellular [24] and cardiac injection [25] as well as incubation with cultured cells as documented in previous studies from other laboratories [16, 25] and ours [8]. This study showed the feasibility of intracellular delivery of exogenous mitochondria through venous administration. Regarding the preservation of mitochondrial structure and function during their systemic application through venous blood in the current study, the sodium-induced inhibition of mitochondrial respiration [26, 27, 28] may be one protective factor since mitochondria do not need to execute respiratory function during the delivery procedures in blood while sodium is present. Figuratively speaking, the inhibition of mitochondrial respiratory function and other related activities by sodium in blood may throw the mitochondria into an inactive state like “hibernation” and this thus protects them from being damaged in extracellular spaces. The mitochondria execute or gain their respiratory function when they localized back to the circumstances or environments similar to intracellular condition, while high potassium is present. This is also the reason for us to use sodium-containing medium for the isolation and preparation of mitochondria in our current and our recent study [8]. Actually, the protocol or procedure employed in our current and our recent study [8] using sodium appears better in maintaining the genetic structure and function of isolated mitochondria [29, 30, 31].

The effectiveness of exogenous mitochondria transplanted before and after the establishment of HPH may suggest a clue for the prevention and therapy against this disease, incurable at the present time. The transplantation of exogenous mitochondria-induced attenuation of acute hypoxic pulmonary vasoconstriction can be explained by the lowered production of reactive oxygen species and the subsequent inhibition of intracellular calcium signaling [8]. The additional explanation for the long-term effects of exogenous mitochondria on reversing chronic hypoxia-inhibited apoptosis, pulmonary vascular remodeling and pulmonary hypertension can be their fusion with endogenous counterpart. The overall mechanisms underlying the effectiveness of exogenous mitochondria *in vivo* may be their intracellular delivery within PASMCs and other types of cells and or their adhesion to the surface of PASMCs (Figure 1).

It is well established in recent studies that mitochondrial DNA escaped to the extracellular space acts as a damage-associated molecular pattern signal and evokes a severe inflammatory response. In the current study, what we administrated into rats via intravenous injection were intact mitochondria, not mitochondrial DNA or mitochondrial fragments/particles. The possibility for DNA escaping from exogenous mitochondria after their administration into animals seems very low. Romani and Ozelkok proved that mitochondria can survive for at least 72 hours at 25°C after isolation as revealed by readily identifiable ultrastructure under electron microscopy and the activities of metabolic and respiratory function supplied with substrate [32]. The survival period of isolated mitochondria can be achieved even after sedimentation and re-suspension and the accelerated metabolic activity appeared to shorten survival [32]. As mentioned above, we used a novel, improved method for the isolation and preparation of mitochondria with medium containing sodium. The sodium is known to inhibit metabolic and respiratory activities of isolated mitochondria *in vitro* [26, 27, 28]. This may therefore provide a protection of exogenous mitochondria from damage and DNA escape during their preparation *in vitro* and their circulating in the blood stream before they gain their function after transplantation into the cells where high potassium is present. Additionally, the delivery of exogenous mitochondria into pulmonary arteries appeared quickly, occurring from ≤ 2-24 hours and reaching a stable level from ~30 hours after their intravenous administration (Figure 1). All these appear to guarantee the survival and integrity of exogenous mitochondria in the blood stream and therefore prevent being broken or escape of their DNA.

Although the rats exposed to chronic hypoxia is one classical model of pulmonary hypertension, pulmonary hypertension can also occur through mechanism(s) independent of hypoxia. It remains unclear whether results of the current study in this chronic hypoxia model will be relevant to any other type(s) of pulmonary hypertension in experimental investigations. Further work is required for the translation of our findings into any human study, e.g. any long term adverse effect from transplanted mitochondria has not been evaluated in the current study. Mitochondria dysfunction is widely involved in pathophysiological processes of diseases in a variety of cells, tissues and organs. It becomes interesting to reveal whether mitochondria transplantation is generally applicable in other organs or tissues and whether it is a valuable approach for the treatment and or prevention of other diseases in experimental models.

## MATERIALS AND METHODS

### Ethical approval

All our studies using Sprague-Dawley (SD) rats were approved by the Institutional Animal Care and Use Committee of Huazhong University of Science and Technology, and performed in accordance with NIH guidelines for the care and use of laboratory animals.

### Anaesthesia and euthanasia

SD rats were irreversibly anesthetized by an intraperitoneal injection of urethane (1.2 g/kg in phosphate-buffered saline, PBS). When the sufficient status of anaesthesia was confirmed by negative paw pinch test, hemodynamic studies were conducted as described below. Alternatively, the femoral arteries and lung tissues were excised quickly from SD rats for the isolation of pulmonary arteries and the subsequent culture of smooth muscle cells, the recovery of mitochondria, immunohistochemical analysis and electron microscopy study.

### Cell culture and mitochondrial preparations

The culture and maintenance of rat smooth muscle cells (SMCs) from explants of the third branches of pulmonary artery and femoral artery [3, 8], and the mitochondrial labeling, preparation from SMCs and their numbering were performed as we recently described in details [8].

### Separation and recovery of transplanted mitochondria

To recover exogenous mitochondria after their transplantation into pulmonary arteries and lung tissues, rats were intravenously injected with 2.25×10^8^/ml DsRed-labeled 6 mg, wet weight)1.3 g, wet weight). processed on a FACS flow cytometer (BD FACSAria II, Becton, Dickinson and Company, USA) for the recovery of the endogenous mitochondria and DsRed-labeled mitochondria using a BD FACSDiva software. The unlabeled mitochondria were used a negative control to set up voltages of forward scatter (FSC) and side scatter (SSC) at 220v and 346v, respectively [8].

### Assessment of mitochondrial respiratory function

The oxygen consumption and respiratory control ratio (RCR) in isolated mitochondria were determined using a Clark-type oxygen electrode system (Oxytherm, Hansatech, UK) and a Mitochondria RCR Assay Kit (Genmed. Arlington, MA, USA). The measurements of mitochondrial membrane potential and H_2_O_2_ production from isolated mitochondria were performed using rhodamine 123 [8] and Amplex UltraRed as described in other lab [33] and ours [8], respectively. The detailed procedures for each of the above measurements were described fully in our recent study [8]. For each separate experiment, triplicate measurements were conducted.

### Hemodynamic studies and administration of mitochondria in rats

SD rats aged in 7-8 weeks and weighed between 180-250 g were randomly assigned into control and treated groups. The rats were housed in an automated airflow chamber (OxyCycler Model A84XOV, Biospherix, Lacona), maintained at 25°C and gassed with 10% O_2_ for 8 hours a day or with room air (21% O_2_) for 4 weeks.

Hemodynamic studies were conducted as fully described in our recent study [3], including continuous monitoring the pressure of pulmonary artery (Ppa) and the pressure of systemic artery (Psa) through catheterizations. The cardiac output was obtained employing the thermodilution technique.

By the end of hemodynamic experiments, pre-warmed saline was introduced into pulmonary circulation through the pulmonary artery catheter and drained from the left atrium to wash out the blood. Then the lungs and heart tissues were removed, and the lungs were fixed with formaldehyde solution (10%) for ≥ 3 days at room temperature. The right ventricular hypertrophy was quantified by weighting the right ventricular free wall (RV) and the left ventricle (LV) together with the septum (S, LV+S). For the analysis of pulmonary morphometry, one sagittal 5 μm-thick section from the top, middle and bottom of each left lung was embedded with paraffin. Then two slides from each section were subjected to Hematoxylin and Eosin Stainings. The medial wall thickness was determined in distal pulmonary arteries with diameters between 50 and 150 μm.

Mitochondria (2.25×10^8^, ~ 2 μg mitochondrial protein) suspended in 1 ml PBS or PBS alone were intravenously via the tail vein injected into rats either every other day during the last 2 weeks of the 4 weeks of exposure to hypoxia, or every other day for 2 weeks after the 4 weeks of exposure to hypoxia. The mitochondria were prepared from ~ 2.5×10^6^ FASMCs at third passage, which primarily cultured from 2-3 rats.

### Liver of Wilson's disease rat model

The Wilson's disease model was generated by feeding copper-loaden in SD rats as reported in other laboratory [34] and ours [8]. The mitochondria in the Wilson's disease rat liver exhibited characteristic ultrastructure with unclear and swelling cristae, increased density of matrix, discrete inclusions as well as round shape as described by other investigators [34] and our own [8], and thus employed in the current study as one tracer to track exogenous mitochondria *in vivo*.

### Immunohistofluorescent staining

24 hours after the administration of DsRed-labeled mitochondria into rats via tail vein (2.25×10^8^), the pulmonary arteries and femoral arteries were isolated from rats and fixed for preparation of sliced sections. To block endogenous peroxidase activity, the sections were pretreated with 3% H_2_O_2_-methanol solution (vol/vol) at room temperature (RT) for 15 min and washed by PBS for at least 3 times, 3 min each. Then the sections were immersed in blocking solution containing 5% fetal bovine serum (BSA, wt/vol) in PBS at RT for 30 min in a humidified chamber. After draining off the blocking solution, the sections were incubated with primary antibody against DsRed (1:100 dilute in 0.5% BSA in PBS, Biovision) in a humidified chamber at 4°C overnight. After washed by PBS for at least 3 times, Cy3-conjugated IgG (1:100 diluted in 0.5% BSA in PBS, Jackson) was applied to the sections and incubated at RT for 60 min in a humidified dark chamber. After washed by PBS for at least 3 times, the sections were incubated with primary antibody against smooth muscle specific α-actin (for pulmonary and femoral arteries, 1:100 diluted in 0.5% BSA in PBS, Abcam) in a humidified dark chamber at 4°C overnight. After washed by PBS for at least 3 times, FITC-conjugated (1:100 diluted in 0.5% BSA in PBS, Jackson) was applied to the sections and incubated in a humidified dark chamber at RT for 60 min. Washed by PBS for 3 times, 5 min each and briefly incubated with 50 μl DAPI for nuclear staining, the sections were rinsed with PBS for 3 times before visual imaging under fluorescent microscope. For each section, all the fields were examined and 4-5 individual, randomly chosen fields of fluorescent images and their corresponding light fields were photographed.

### Electron microscopy and ultrastructural study

The lung tissues and pulmonary arteries from at least 3 rats for each group were fixed following our modification of Zischka's method [34] and our recent work [8]. The thin sections were made with an ultramicrotome, stained by OsO4, and examined on a transmission electron microscope (FEI Tecnai G2 20 TWIN) using an Olympus CCD (Cantega G2) and software (Cantega G2) with an acceleration voltage of 80 kV, filament voltage preset at 19V, emission current preset at 10 mA, live image acquisition of 100 ms, capture exposure of 1600 ms and camera gain of 80. To visualize ascorbate peroxidase (APEX)-labeled mitochondria [8, 9, 10], tissues and pulmonary arteries were overlaid with H_2_O_2_ and 3,3′-diaminobenzidine to allow APEX-catalyzed polymerization for 25 minutes before staining with OsO4.

### Statistical analysis

The results were shown in means ± standard errors. For comparisons between two or multiple groups, the Student's t-test and One-Way ANOVA analysis were conducted, respectively. A significant difference was set at *p* < 0.05.

## References

[1] Kuhr FK, Smith KA, Song MY, Levitan I, Yuan JX (2012). New mechanisms of pulmonary arterial hypertension: role of Ca^2+^ signaling. American Journal of Physiology-Heart and Circulatory Physiology.

[2] Marsboom G, Toth PT, Ryan JJ, Hong Z, Wu X, Fang YH, Thenappan T, Piao L, Zhang HJ, Pogoriler J, Chen Y, Morrow E, Weir EK (2012). Dynamin-related protein 1-mediated mitochondrial mitotic fission permits hyperproliferation of vascular smooth muscle cells and offers a novel therapeutic target in pulmonary hypertension. Circulation Research.

[3] Zhang J, Zhou J, Cai L, Lu Y, Wang T, Zhu L, Hu Q (2012). Extracellular calcium-sensing receptor is critical in hypoxic pulmonary vasoconstriction. Antioxidants & Redox Signaling.

[4] Leach RM, Sheehan DW, Chacko VP, Sylvester JT (2000). Energy state, pH, and vasomotor tone during hypoxia in precontracted pulmonary and femoral arteries. American Journal of Physiology-Lung Cellular and Molecular Physiology.

[5] Zoer B, Cogolludo AL, Perez-Vizcaino F, De Mey JG, Blanco CE, Villamor E (2010). Hypoxia sensing in the fetal chicken femoral artery is mediated by the mitochondrial electron transport chain. American Journal of Physiology-Regulatory Integrative and Comparative Physiology.

[6] Firth AL, Gordienko DV, Yuill KH, Smirnov SV (2009). Cellular localization of mitochondria contributes to Kv channel-mediated regulation of cellular excitability in pulmonary but not mesenteric circulation. American Journal of Physiology-Lung Cellular and Molecular Physiology.

[7] Michelakis ED, Hampl V, Nsair A, Wu X, Harry G, Haromy A, Gurtu R, Archer SL (2002). Diversity in mitochondrial function explains differences in vascular oxygen sensing. Circulation Research.

[8] Zhou J, Zhang J, Lu Y, Huang S, Xiao R, Zeng X, Zhang X, Li J, Wang T, Li T, Zhu L, Hu Q (2016). Mitochondrial Transplantation Attenuates Hypoxic Pulmonary Vasoconstriction. Oncotarget.

[9] Martell JD, Deerinck TJ, Sancak Y, Poulos TL, Mootha VK, Sosinsky GE, Ellisman MH, Ting AY (2012). Engineered ascorbate peroxidase as a genetically encoded reporter for electron microscopy. Nature Biotechnology.

[10] Rhee HW, Zou P, Udeshi ND, Martell JD, Mootha VK, Carr SA, Ting AY (2013). Proteomic mapping of mitochondria in living cells via spatially restricted enzymatic tagging. Science.

[11] Davies P, Burke G, Reid L (1986). The structure of the wall of the rat intraacinar pulmonary artery: an electron microscopic study of microdissected preparations. Microvascular Research.

[12] Michel RP, Hu F, Meyrick BO (1995). Myoendothelial junctional complexes in postobstructive pulmonary vasculopathy: a quantitative electron microscopic study. Experimental Lung Research.

[13] Townsley MI (2012). Structure and composition of pulmonary arteries, capillaries, and veins. Comprehensive Physiology.

[14] Wagenvoort CA, Dingemans KP (1985). Pulmonary vascular smooth muscle and its interaction with endothelium. Morphologic considerations. Chest.

[15] Hameed AG, Arnold ND, Chamberlain J, Pickworth JA, Paiva C, Dawson S, Cross S, Long L, Zhao L, Morrell NW, Crossman DC, Newman CM, Kiely DG (2012). Inhibition of tumor necrosis factor-related apoptosis-inducing ligand (TRAIL) reverses experimental pulmonary hypertension. Journal of Experimental Medicine.

[16] Kitani T, Kami D, Matoba S, Gojo S (2014). Internalization of isolated functional mitochondria: involvement of macropinocytosis. Journal of Cellular and Molecular Medicine.

[17] Islam MN, Das SR, Emin MT, Wei M, Sun L, Westphalen K, Rowlands DJ, Quadri SK, Bhattacharya S, Bhattacharya J (2012). Mitochondrial transfer from bone-marrow-derived stromal cells to pulmonary alveoli protects against acute lung injury. Nature Medicine.

[18] Spees JL, Olson SD, Whitney MJ, Prockop DJ (2006). Mitochondrial transfer between cells can rescue aerobic respiration. Proceedings of The National Academy of Sciences of The United States of America.

[19] Chen JK, Shih MH, Peir JJ, Liu CH, Chou FI, Lai WH, Chang LW, Lin P, Wang MY, Yang MH, Yang CS (2010). The use of radioactive zinc oxide nanoparticles in determination of their tissue concentrations following intravenous administration in mice. Analyst.

[20] Gipps EM, Arshady R, Kreuter J, Groscurth P, Speiser PP (1986). Distribution of polyhexyl cyanoacrylate nanoparticles in nude mice bearing human osteosarcoma. Journal of Pharmaceutical Sciences.

[21] Sonavane G, Tomoda K, Makino K (2008). Biodistribution of colloidal gold nanoparticles after intravenous administration: effect of particle size. Colloids and Surfaces B-Biointerfaces.

[22] Liu D, Yan Z, Minshall RD, Schwartz DE, Chen Y, Hu G (2012). Activation of calpains mediates early lung neutrophilic inflammation in ventilator-induced lung injury. American Journal of Physiology-Lung Cellular and Molecular Physiology.

[23] Mirza MK, Sun Y, Zhao YD, Potula HH, Frey RS, Vogel SM, Malik AB, Zhao YY (2010). FoxM1 regulates re-annealing of endothelial adherens junctions through transcriptional control of beta-catenin expression. Journal of Experimental Medicine.

[24] King MP, Attardi G (1988). Injection of mitochondria into human cells leads to a rapid replacement of the endogenous mitochondrial DNA. Cell.

[25] Masuzawa A, Black KM, Pacak CA, Ericsson M, Barnett RJ, Drumm C, Seth P, Bloch DB, Levitsky S, Cowan DB, McCully JD (2013). Transplantation of autologously derived mitochondria protects the heart from ischemia-reperfusion injury. American Journal of Physiology-Heart and Circulatory Physiology.

[26] MacDonald MJ (1984). The use of calcium uptake by small amounts of mitochondria from pancreatic islets to study mitochondrial respiration: the effects of diazoxide and sodium. Biochemistry International.

[27] Vlessis AA, Widener LL, Bartos D (1990). Effect of peroxide, sodium, and calcium on brain mitochondrial respiration *in vitro*: potential role in cerebral ischemia and reperfusion. Journal of Neurochemistry.

[28] Babsky A, Doliba N, Doliba N, Savchenko A, Wehrli S, Osbakken M (2001). Na^+^ effects on mitochondrial respiration and oxidative phosphorylation in diabetic hearts. Experimental Biology and Medicine (Maywood).

[29] Fernández-Vizarra E, López-Pérez MJ, Enriquez JA (2002). Isolation of biogenetically competent mitochondria from mammalian tissues and cultured cells. Methods.

[30] Fernández-Vizarra E, Ferrín G, Pérez-Martos A, Fernández-Silva P, Zeviani M, Enríquez JA (2010). Isolation of mitochondria for biogenetical studies: An update. Mitochondrion.

[31] Satori CP, Kostal V, Arriaga EA (2012). Review on recent advances in the analysis of isolated organelles. Analytica Chimica Acta.

[32] Romani RJ, Ozelkok S (1973). “Survival” of mitochondria *in vitro*: physical and energy parameters. Plant Physiology.

[33] Quinlan CL, Treberg JR, Perevoshchikova IV, Orr AL, Brand MD (2012). Native rates of superoxide production from multiple sites in isolated mitochondria measured using endogenous reporters. Free Radical Biology and Medicine.

[34] Zischka H, Lichtmannegger J, Schmitt S, Jägemann N, Schulz S, Wartini D, Jennen L, Rust C, Larochette N, Galluzzi L, Chajes V, Bandow N, Gilles VS (2011). Liver mitochondrial membrane crosslinking and destruction in a rat model of Wilson disease. Journal of Clinical Investigation.

